# Supplementary material for: Suppressed prefrontal neuronal firing variability and impaired social representation in IRSp53-mutant mice
Source: eLife. 2022 Nov 1;11:e74998. doi: 10.7554/eLife.74998 (PMC9662818; doi:10.7554/eLife.74998)
Supplement: Transparent reporting form [file elife-74998-transrepform1.docx]

***eLife’s* transparent reporting form**

We encourage authors to provide detailed information *within their submission* to facilitate the interpretation and replication of experiments. Authors can upload supporting documentation to indicate the use of appropriate reporting guidelines for health-related research (see [EQUATOR Network](http://www.equator-network.org/%20)), life science research (see the [BioSharing Information Resource](https://biosharing.org/)), or the [ARRIVE guidelines](http://www.plosbiology.org/article/info:doi/10.1371/journal.pbio.1000412) for reporting work involving animal research. Where applicable, authors should refer to any relevant reporting standards documents in this form.

If you have any questions, please consult our Journal Policies and/or contact us: [editorial@elifesciences.org](mailto:editorial@elifesciences.org).

**Sample-size estimation**

The conclusion of our study was derived from **1) animal behavior data**, **2) single-unit recording data** and **3) slice electrophysiology data**. We did not use any explicit power analysis, but instead, referred to the previous literatures of this field for the generally-accepting animal/unit sample sizes. For example:

- In a literature of this field, 295 units from 5 WT mice and 409 units from 5 Shank2-KO mice were used for initial genotypic comparisons, whereas 163 units from 4 EYFP Shank2-KO PV-cre mice and 364 units from 9 ChR2 Shank2-KO PV-cre mice were used further rescue experiment analysis (Lee et al., 2021).

- In another literature of this field, 194 units from 6 mice were used for initial WT analysis, whereas 159 units from 5 WT mice and 131 units from 6 Cntnap-KO mice were used for further genotypic comparisons (Levy et al., 2019).

**1) Animal behavior data:** We used at least 6 animals for each group ([WT] n = 6, [IRSp53-KO] n = 8) and performed 12 repeated recordings for each animal with 3-day intervals.

**2) Single-unit recording data:** We collected neural data from 220–270 units for each group ([WT] n = 233, [IRSp53-KO] n = 258) from 6 WT and 8 IRSp53-KO mice across 12 recordings.

Information on the sample size are stated in the figure legends and supplementary file 1 and 2.

Lee, E., Lee, S., Shin, J.J., Choi, W., Chung, C., Lee, S., Kim, J., Ha, S., Kim, R., Yoo, T.*, et al.* (2021). Excitatory synapses and gap junctions cooperate to improve Pv neuronal burst firing and cortical social cognition in Shank2-mutant mice. Nat Commun *12*, 5116.

Levy, D.R., Tamir, T., Kaufman, M., Parabucki, A., Weissbrod, A., Schneidman, E., and Yizhar, O. (2019). Dynamics of social representation in the mouse prefrontal cortex. Nat Neurosci *22*, 2013-2022.

**3) Slice electrophysiology data**: We collected intrinsic property data from at least 3 animals for each group ([WT] n = 24 neurons from 3 mice, [WT+mem] n = 18, 3, [KO] n = 23, 3, [KO+mem] n = 17, 3).

**Replicates**

Data from the single unit recording experiments were collected from 3 separate cohorts. The animal number for each cohort was determined by the available number of littermates (of same age, size, and gender) at the time of experiment. Fairly balanced numbers of WT and IRSp53-KO mice were used for each cohort, and the raw data was harvested by one researcher in a controlled time window (a total of 12 recordings with 3-day intervals) and experimental setting. Detailed information on the number of units collected from individual mice from individual cohorts is indicated in supplementary file 1.

For each mouse, in order to obtain sufficient number of units, recordings were technically replicated 12 times. Sniffing trials with the duration of < 1 sec and inter-trial interval of < 2 sec, as well as, in-zone (and center zone) trials with the duration of < 0.5 sec and inter-trial interval of < 0.5 sec were excluded from analysis. Valid proximal and distal trials with the duration of < 1 sec were excluded from analysis. Units with missing valid sniffing and in-zone trials for any of the six targets (left and right for the E-E session, social and object for the first and second S-O sessions) were excluded from analysis. Units with mean firing rate of < 0.5 Hz were excluded for all firing rate analyses except for the initial baseline firing rate analysis in Figure 2. Detailed exclusion criteria are mentioned in the Materials and Methods section.

**Statistical reporting**

Explanations on the statistical analysis are provided in the Materials and Methods section.

The n numbers and statistical tests used are stated in the figure legends. The dispersion and precision measures used for bar graphs (SEM), box and whisker plots (median, interquartile range, 2.5 and 97.5 percentile), and volcano plots (median, interquartile range) are stated in the Materials and Method section, or, otherwise, stated in the figure legends.

Detailed statistical information (exact p-values and summary statistics) and raw data is organized in supplementary file 2 and source files, respectively.

**Group allocation**

Behavior data and single-unit data are allocated into two groups (WT and IRSp53-KO) based on genotype. Electrophysiological data are allocated into four groups (WT, IRSp53-KO, WT+memantine, IRSp53-KO+memantine) based on genotype and memantine (drug) presence.

**Additional data files (“source data”)**

Source data files for all figures (except Figure 1 – Figure Supplement 1, Figure 3, Figure 3 – Figure Supplement 2) are organized in separate source files.
